# Is this a “Fettecke” or just a “greasy corner”? About the capability of laypersons to differentiate between art and non-art via object's originality

**DOI:** 10.1068/i0664

**Published:** 2014-11-28

**Authors:** Manuela Haertel, Claus-Christian Carbon

**Affiliations:** Department of General Psychology and Methodology, University of Bamberg, Bamberg, Bavaria, Germany; e-mail: manuela-gertraud.haertel@stud.uni-bamberg.de; Department of General Psychology and Methodology, University of Bamberg, Bamberg, Bavaria, Germany; and Bamberg Graduate School of Affective and Cognitive Sciences (BaGrACS), Bamberg, Germany; e-mail: ccc@experimental-psychology.com

**Keywords:** empirical aesthetics, visual art, context, aesthetic appreciation, kitsch, originality, expertise, innovativeness, contemporary art

## Abstract

Which components are needed to identify an object as an artwork, particularly if it is contemporary art? A variety of factors determining aesthetic judgements have been identified, among them stimulus-related properties such as symmetry, complexity and style, but also person-centred as well as context-dependent variables. We were particularly interested in finding out whether laypersons are at all able to distinguish between pieces of fine art endorsed by museums and works not displayed by galleries and museums. We were also interested in analysing the variables responsible for distinguishing between different levels of artistic quality. We ask untrained (Exp.1) as well as art-trained (Exp.2) people to rate a pool of images comprising contemporary art plus unaccredited objects with regard to preference, originality, ambiguity, understanding and artistic quality. Originality and ambiguity proved to be the best predictor for artistic quality. As the concept of originality is tightly linked with innovativeness, a property known to be appreciated only by further, and deep, elaboration (Carbon, 2011 *i-Perception, 2*, 708–719), it makes sense that modern artworks might be cognitively qualified as being of high artistic quality but are meanwhile affectively devaluated or even rejected by typical laypersons—at least at first glance.

## 1 Introduction

When Joseph Beuys' famous artwork “Fettecke”^1^ (1982; Engl. “greasy corner”) was nearly destroyed by a diligent facility manager in 1986, a vivid societal debate emerged on what an artwork is about and how such a work is defined in modern times. “Fettecke” obviously polarised attending beholders as many people clearly identified it as a great work of contemporary art whereas others defamed it as just being an object made of greasy substance without any link to art at all.

Whereas many laypersons seemingly reject some works of contemporary art, it has not been scientifically investigated whether laypersons are at all able to assess artistic quality. Although a series of studies exist that investigate the role of stimulus symmetry, complexity, familiarity, fluency, artistic style and so-called good Gestalts on aesthetic appreciation and evaluation (e.g. Augustin, Carbon, & Wagemans, 2012a), the categorisation of objects as artworks or not has not been addressed so far. We only know that context triggers and modifies aesthetic appreciation (Carbon & Jakesch, 2013; Leder Belke, Oeberst, & Augustin, 2004), for instance when inspecting an unfamiliar object in the context of a museum or art gallery, we assign more aesthetic quality (e.g. more pleasantness, more interestingness) to the targeted entity (Locher, Smith, & Smith, 2001). Additionally, context factors have been revealed by means of putative authenticity (Wolz & Carbon, 2014) or the specific entitling of artworks (Leder, Carbon, & Ripsas, 2006; Millis, 2001). The aforementioned shortlist of possible influences and the complex interplay between such aspects and further factors such as personality traits, expertise or interest show the meaningfulness of this topic.

To form a judgement, we have to mentally represent and evaluate the assessed piece of art. Part of the perception of an object to be evaluated is the situational context within an episode (e.g. the encoding specificity, Tulving & Thomson, 1973). The episodic context can be regarded as a kind of scale, embedded into the object which is to be evaluated. As such, the aesthetic judgement process combines bottom-up (analysis of the object) and top-down sub-processes (e.g. by the situation, but also by the level of expertise) (see Carbon & Jakesch, 2013; Leder et al., 2004). A judgement highly depends on the category system of individuals. These determine which information is retrieved, processed and available in the mental representation, the mental model. This idea was propagated specifically for aesthetics by Martindale's (1984, 1988) cognitive theory based on the theory of semantic networks (Quillian, 1968). A semantic network represents semantic relations between concepts, as a form of knowledge representation. The retrieval of knowledge occurs through the activation of a node. This activation is called spreading activation (Collins & Loftus, 1975). Accordingly, an object is perceived as an aesthetic target object if a combination of specific nodes is activated in the network (cf. Faerber, Leder, Gerger, & Carbon, 2010).

In the present study, we used the same context for evaluating all stimuli. As this context did not contain any indication of a typical art environment such as a museum, participants were referred to object-related properties. One such object-related variable which was identified as being important, at least in contemporary art, is *ambiguity*—a quality that offers specific and distinct interpretations of the object. As the processing of aesthetic stimuli was described as a kind of problem solving process (Tyler, 1999), ambiguity and partly the resolving of such ambiguities with the by-product of understanding parts of the meaning of an artwork seems particularly important. The effort of resolving such problems with subsequent better understanding of an artwork might be a part of the pleasure that emerges from a deeper aesthetic experience (Russell, 2003). Biederman and Vessel (2006) explain this on a neural level: The greater the amount of interpretable information, the more activity is possible in the visual association areas and therefore the more perceptual pleasure is produced for the viewer. As pointed out only recently, ambiguity is indeed a characteristic of many artworks (Jakesch & Leder, 2009; Muth & Carbon, 2013; Muth, Pepperell, & Carbon, 2013). Mamassian (2008) highlighted that ambiguity in the visual arts is special as the perceiver has no particular task when inspecting artworks. Therefore, the perceiver does not suffer negative consequences as a result of not being able to resolve the ambiguity in the artwork (but see Cupchik, 1992).

Besides ambiguity, laypersons can describe artworks on the basis of a series of other variables. Augustin and colleagues revealed that each different aesthetic domain such as visual art versus film and music has its own distinct pattern of relevant aesthetic concepts (Augustin et al., 2012a). They showed that when laypersons are asked to describe works of art they not only used relatively undifferentiated concepts like “beautiful” (cf. Jacobsen, Buchta, Köhler, & Schröger, 2004), but also employed terms with strong affective associations reflected by words like “wonderful” and clear cognitive associations such as “originality” (Augustin, Wagemans, & Carbon, 2012b)

In the present paper, we aimed to find out which variables are mainly responsible for assessing the “artistic quality” of ambiguous objects in order to have an idea of what fuels the classification of any aesthetic object into art or non-art. Previous research demonstrated that contextual information has an important impact on aesthetic evaluation, but here we focused on which key variables the aesthetic evaluation of ambiguous objects is based when contextual information is missing. Furthermore, we investigated whether laypersons and art experts can see a difference between the objects and whether factors other than beauty may reflect artistic quality.

## 2 Experiment

### 2.1 Method

#### 2.1.1 Participants

Seventeen persons (12 female, 5 male) aged between 19 and 33 years (*M* = 24.1 years, *SD* = 4.2) participated in the experiment, all of whom were students of the University of Bamberg. They had normal or corrected-to-normal vision confirmed by the Snellen Eye Chart test; furthermore, normal colour vision was assured by a short version of the Ishihara Colour Test. All participants were naïve about modern art as they all lacked special training in the arts. We verified the notion of being laypersons by asking them about the possession of art reference books and about their experience with art exhibitions as in Leder et al. (2006).

#### 2.1.2 Stimuli

We used pictures of ambiguous objects as they are not clearly identifiable and solvable entities for which we clearly need cognitive effort to infer meaning—in this regard they are also not easily processed on a perceptual level due to basic mechanisms such as fluency (see Reber, Schwarz, & Winkielman, 2004; see also Albrecht & Carbon, 2014). We retrieved 213 colour photographs of various objects, 134 contemporary art objects (e.g., Salvador Dali's “Lobster-Telephone” or Tracy Emin's “My Tent” installation) and 79 unaccredited objects (“everyday objects”). Both art objects and everyday objects were selected during extensive Internet research. We took care to secure a comparable degree of ambiguity of copies of both object categories. The criterion of *ambiguity* is characterised in that the chosen art and everyday objects have no clear meaning and function and can thus be arbitrarily described as an “art object” or as an everyday object (“non-art object”). At first appearance, both object categories are very similar, so that the distinction between them is based on formal criteria. We pre-categorised the objects as art objects, when they were exhibited in a prestigious museum or produced by a renowned artist. If an object did not satisfy the formal criteria, it was considered as a non-art object.

#### 2.1.3 Procedure

All images were repeatedly shown over five blocks, for each block all images were fully randomised again and again. Participants were asked to rate one image after another on 7-point Likert-scales (1 = *not at all* to 7 = *very strong*) regarding one of the following five dimensions (one dimension consistently during each block): 1) *Preference*, 2) *originality*, 3) *ambiguity*, 4) *understanding* and 5) *artistic quality*. The block order was kept constant across all participants. Participants were asked to respond as quickly and accurately as possible, so following their first impression. After each block, participants made use of a small break; the whole study lasted approximately 90 min.

### 2.2 Results

**Figure 1.**
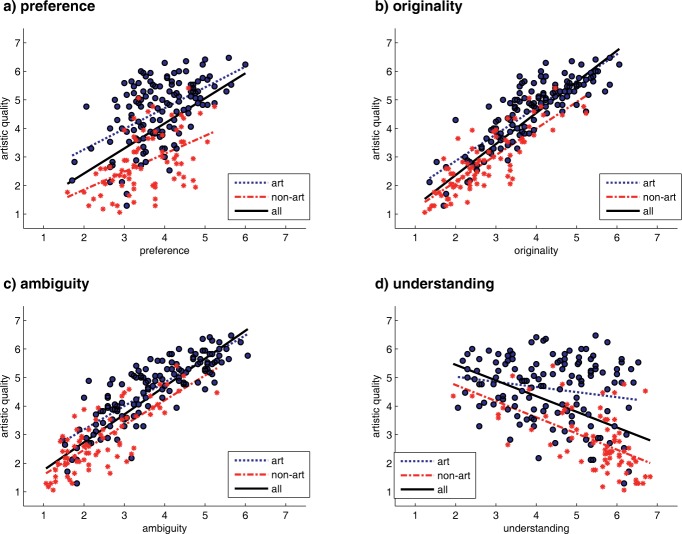
Bivariate diagrams showing the relationship between the dimensions *artistic quality* and (a) *preference,* (b) *originality,* (c) *ambiguity,* (d) *understanding,* split by the categories of art (blue solid dots) and non-art objects (red asterisks). Dotted lines indicate linear fits for each category separately; solid lines show the overall linear fits when taking both categories together.

As seen in Figure 1, all dimensions correlated significantly with artistic quality. In contrast to the empirical findings, which we have mentioned above, *originality* (*r* = 0.87, *p*<.0001) and *ambiguity* (*r* = 0.87, *p*<.0001) were positively correlated with artistic quality, as was *preference* (*r* = 0.51, *p*<.0001) although to a lesser extent. *Understanding*, on the other hand, was negatively correlated with the dimension *artistic quality* (*r* = −0.48, *p*<.0001).

A compatible pattern of results emerged when conducting a multiple regression analysis with *artistic quality* as dependent variable (see Table 1). Once again, *preference* played only a minor part in explaining *artistic quality* (β = 0.13). Much more prominent as predictor was *ambiguity* (β = 0.29) and, even more, *originality* (β = 0.47). As in the single bivariate analyses, *understanding* was clearly negatively associated with *artistic quality* (β = −0.34). The overall explained variance of the whole linear regression model was 89% (*R* = .948, *N* = 213, *p*<.0001), so *artistic quality* could be substantially predicted by the targeted four variables.

**Table 1.** Experiment 1 (laypersons): Results of the multiple regression analysis for *artistic quality* as dependent variable.

Predictors	*B*	SE *B*	β	*t*	*p*
Preference	0.21	0.05	0.13	3.79	<.0001
Originality	0.58	0.08	0.47	7.17	<.0001
Ambiguity	0.32	0.06	0.29	5.05	<.0001
Understanding	− 0.39	0.02	− 0.34	− 14.05	<.0001

**Figure 2.**
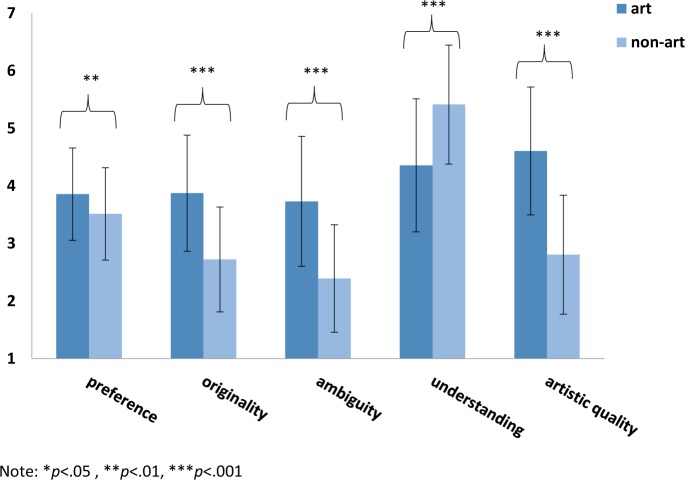
Comparison of mean values on the variables *preference*, *originality*, *ambiguity*, *understanding* and *artistic quality* for art vs non-art objects. Error bars indicate ±1 SEM. Asterisks show significant differences between art and non-art objects.

We also found significant differences between all ratings of art and non-art objects (Figure 2), analysed by two-tailed *t*-tests: *preference*: *t*(211) = 3.00, *p* = .003, *d* = 0.43; *originality*: *t*(211) = 8.32, *p*<.0001, *d* = 1.19; *ambiguity*: *t*(211) = 8.90, *p*<.0001, *d* = 1.24; *understanding*: *t*(211) = −6.69, *p*<.0001, *d* = 0.95; *artistic quality*: *t*(211) = 11.72, *p*<.0001, *d* = 1.67.

Experiment 1 provides insights into evaluating objects of artistic quality by using a broad variety of stimulus material, consisting of art and non-art, and thus unaccredited, objects. Of all analysed variables (*preference*, *originality*, *ambiguity*, *understanding*), *originality* was the best predictor for *artistic* *quality*, while *preference* only showed a relatively weak, but still significant, association. In addition, *artistic quality* was positively associated with *ambiguity* but negatively with *understanding*. Furthermore, we demonstrated that participants performed quite impressively in differentiating between art and non-art objects in an implicit way. This is quite astonishing as all participants were naïve about art, particularly contemporary art.

## 3 Experiment 2

To check whether the correlations we found in Experiment 1 were valid even for art-trained people, we ran Experiment 2. The research about expertise has a long tradition in psychology and has been extensively studied in cognitive research fields such as chess playing (Simon & Chase, 1973) as well as in more perceptual research fields such as face processing (Schwaninger, Carbon, & Leder, 2003). Expertise is seen as a very high level of domain-specific knowledge with specific highly trained skills which are accumulated through experience and duration of deep elaboration (Van den Bos, 2007). The image perception of art-trained viewers and non-trained viewers was investigated in several studies, for instance, demonstrating that art-trained and non-trained viewers judge artworks in a qualitatively different way. It has been shown for example that for art-trained viewers, complexity (Silvia, 2006) and originality (Hekkert & Van Wieringen, 1996) are related to artistic quality. In comparison to art-trained viewers who process artworks more on a subordinate level (Belke, Leder, Harsanyi, & Carbon, 2010), non-trained viewers rather look at obvious details on the mere content of an artwork or at how the artwork was primarily made (Cupchik & Geboyts, 1988). Augustin and Leder (2006) dealt with art expertise relating to contemporary art. They found that experts process artworks more in relation to style, whereas non-experts do so using personal criteria such as feelings. Vogt and Magnussen (2007) provided further evidence for different viewing strategies of art-trained and non-trained beholders: Non-trained viewers spend more time on areas with recognisable objects and human features than art-trained viewers do. The aim of Experiment 2 was to analyse whether art-trained people differ in the variables that predict their assessment of artistic quality of our target objects compared with the laypersons in Experiment 1.

### 3.1 Method

#### 3.1.1 Participants

We recruited twenty participants (9 female, 11 male), all of whom were students at Cardiff School of Art and Design with intense general fine art training (aged from 19 to 38 years, *M* = 24.8 years; *SD* = 5.8). All had normal or corrected-to-normal vision, again assured by standard vision and colour vision tests as described in Experiment 1.

#### 3.1.2 Stimuli

Based on the results of Experiment 1, 80 images (40 art objects/ 40 non-art objects) were selected from the pool of 213 images from Experiment 1 to reduce the duration time of the experiment. As known from Experiment 1, participants evaluated some images very similarly on the target dimensions, so we dropped such redundant items without losing much of the variety of the entire set for all the targeted dimensions of *preference*, *originality*, *ambiguity*, *understanding* and *artistic quality.*

#### 3.1.3 Procedure

The procedure was the same as in Experiment 1.

### 3.2 Results

We found a highly compatible pattern of relationships between our variables as in Experiment 1. All used variables correlated significantly with *artistic quality* as can be seen in Table 2.

**Table 2.** Correlations between the five dimensions (*preference, originality, ambiguity, understanding* and *artistic quality*) for art-trained (Experiment 2) vs. non-trained participants (Experiment 1).

		Preference	Originality	Ambiguity	Understanding	Artistic quality
Preference	Art-trained	1	.39***	.48***	.13	.68***
	Non-trained	1	.65***	.48***	.17*	.51***
Originality	Art-trained	.39***	1	.87***	−.59***	.78***
	Non-trained	.65***	1	.90***	−.18*	.87***
Ambiguity	Art-trained	.48***	.87***	1	−.60***	.83***
	Non-trained	.48***	.90***	1	−.30***	.87***
Understanding	Art-trained	.13	−.59***	−.60***	1	−.38***
	Non-trained	.17*	−.18*	−.30***	1	−.48***
Artistic quality	Art-trained	.68***	.78***	.83***	−.38***	1
	Non-trained	.51***	.87***	.87***	−.48***	1

Note: *p < .05

** *p* < .01

*** *p* < .001.

When conducting a multiple regression analysis with *artistic quality* as dependent variable (see Table 3), we observed an interesting specific predicting role of *preference* (β = 0.40) and *ambiguity* (β = 0.36): In contrast to laypersons, experts based their assessments of artistic quality more on their own personal preference instead of the probably ultimate criterion for an artwork of being “original” (Wolz & Carbon, 2014). This was quite surprising and could mean experts place *more* trust in their gut feelings (here: trusting in the affective value of their own liking) than cognitively analysing the object by assessing originality. The overall explained variance of the whole was again very high at 82% (*R* = .909, *N* = 80, *p*<.0001), so *artistic quality* again could be substantially predicted by three out of four variables—only *understanding* did not significantly contribute to the whole model. This might be interpreted as further evidence that art-trained people do *not* use typical cognitive processes such as analysis of their level of understanding, but based their evaluations rather more on gut feelings and intuition. Hence, the idea of explicitly trying to understand artworks by reading the “hidden message” might be a particular mode of cognitive processing prevalent in laypersons but not experts.

**Table 3.** Experiment 2 (art-trained persons): Results of the multiple linear regression analysis for *artistic quality* as dependent variable.

	*B*	SE *B*	β	*t*	*p*
Preference	0.56	0.09	0.40	5.89	<.0001
Originality	0.24	0.08	0.28	2.85	.006
Ambiguity	0.34	0.11	0.36	3.097	.003
Understanding	− 0.06	0.09	− 0.05	− 0.65	.518 (ns)

By using two-tailed *t*-tests, we again found good performance in telling apart art and non-art objects across all dimensions: *Preference*: *t*(78) = 1.88, *p* <.064, *d* = 0.44, *originality*: *t*(78) = 5.54, *p* <. 0001, *d* = 1.26, *ambiguity*: *t*(78) = 6.43, *p* <. 0001, *d* = 1.47, *understanding*: *t* (78) = −3.78, *p* <.0001, *d* = 0.87 and *artistic quality*: *t*(78) = 5.37, *p* <. 0001, *d* = 1.22.

## 4 General discussion

The present study provides further insights into the multidimensionality of variables underlying the assessment of artistic quality. In our experiment, we employed a broad variety of stimulus material, consisting of art and (unaccredited) non-art objects. We used two very different groups of participants: in Experiment 1 laypersons and in Experiment 2 art-trained persons assessing the material.

For the laypersons we found that of all analysed variables (*preference*, *originality*, *ambiguity*, *understanding*), *originality* was the best predictor for *artistic quality*, while *preference* only showed moderate associations. In addition, *artistic quality* was positively associated with *ambiguity* and negatively associated with *understanding.* Furthermore, we demonstrated that even laypersons could already easily differentiate between art and non-art objects in an implicit way—this is quite astonishing for the group of participants who were naïve to art, particularly naïve to contemporary art. Based on these data, it can be assumed that contemporary art objects do not need to be fully understood in order to be considered as “high art,” but rather by their originality and ambiguity—the data can probably even be interpreted in such a way that some items which cannot be understood very well are qualified as being art particularly *because* they are not easy on the mind. The results also show an additional aspect: Although we could show that art objects were liked more than non-art objects on average, we also revealed that other predictors were much more influential than the variable preference which has been notoriously assumed to be the most relevant variable in aesthetics. This reflects findings by Augustin and colleagues (e.g. Augustin et al., 2012a, 2012b) that showed that “beauty” or linked concepts such as attractiveness are far from being the most influential for appreciating and processing art.

For the art-trained experts whom we tested in Experiment 2, we found a similar pattern among the key variables as we did for the laypersons. Again, *preference* was not the best predictor for *artistic quality* compared to *originality* and *ambiguity*. *Understanding,* interestingly, although again showing a negative correlation with *artistic quality*, did not reach significance as a predictor in the multiple regression model. Experts seem to assess artworks more through personal preference than through any other variable which has been addressed in our study. As already described by Leder et al. (2004) in the model of aesthetic experience, art-trained persons have more knowledge and experience about art, which has an impact on implicit processing as well. So, it seems on the one hand that art experts perceive art rather implicitly and automatically, relying more on their gut feeling to identify an art-object—gut feelings of course in the sense of highly trained processes by processing a huge number of objects combined with extensive semantic knowledge. On the other hand, laypersons tend to use more explicit and analytical ways of evaluating ambiguous objects—a phenomenon well known in the context of art exhibitions where laypersons continually ask for meaning of the art and often long for the dissolution of ambiguities. Evidence for this can also be observed in the data pattern of the present experiments, particularly in the difference of the ratings of understanding across both studies: For the layperson-model, understanding predicts artistic quality together with the other utilized dimensions, but these effects could not be shown for the art-trained experts. There might be several reasons for this dissociate finding. Artists might understand artworks much better and deeper, but in an implicit way—on an explicit level, understanding is less important for them. Alternatively, art-trained persons might better cope with lack of understanding or not solving the ambiguity, because other aspects such as ambiguity or originality are more important in their evaluations. Leder, Gerger, Dressler and Schabmann (2012) have investigated the aesthetic appreciation of classic, abstract and modern artworks. They found also that understanding plays a greater role in the valuation of art by non-art trained persons, while understanding always depends on the style of the artwork. Additionally, Van den Cruys and Wagemans (2011) reported that less understanding (prediction error) of an artwork can even cause a deeper elaboration of art, because observers have to spend more time finding the meaning in art. Accordingly, Pepperell (2011) assumed that the mental effort a person has to invest in order to recognise the content of a piece of art has a positive influence on aesthetic appreciation.

To sum up, art experts seem to rely on their gut feelings or intuition when evaluating art while laypersons try to understand the hidden message of the art object. However such experts' gut feelings might actually be very reliable and valid heuristics.

## Footnotes

1 “Fettecke” was a conceptual artwork by the German artist and art theorist Joseph Beuys (1921–1986) installed in a corner of his “Atelier 3” of the Arts Academy in the city of Düsseldorf. It consisted of a solid pile of 5 kg of butter directly applied to the wall, intended to work as a permanent demonstration object.
